# Adenovirus-mediated siRNA targeting Bcl-xL inhibits proliferation, reduces invasion and enhances radiosensitivity of human colorectal cancer cells

**DOI:** 10.1186/1477-7819-9-117

**Published:** 2011-10-04

**Authors:** Jinsong Yang, Ming Sun, Aiping Zhang, Chengyu Lv, Wei De, Zhaoxia Wang

**Affiliations:** 1 Department of Oncology, The Affiliated Nanjing First Hospital, Nanjing Medical University, Nanjing, Jiangsu, People's Republic of China; 2 Department of Biochemistry and Molecular Biology, Nanjing Medical University, Nanjing, Jiangsu, People's Republic of China; 3 Department of Cardio-Thoracic Surgery, The Affiliated Nanjing First Hospital, Nanjing Medical University, Nanjing, Jiangsu, People's Republic of China; 4 Department of General Surgery, The Affiliated Nanjing First Hospital, Nanjing Medical University, Nanjing, Jiangsu, People's Republic of China; 5 Department of Oncology, The Second Affiliated Hospital, Nanjing Medical University, Nanjing, Jiangsu, People's Republic of China

## Abstract

**Introduction:**

Bcl-xL, an important member of anti-apoptotic Bcl-2 family, plays critical roles in tumor progression and development. Previously, we have reported that overexpression of Bcl-xL was correlated with prognosis of colorectal cancer (CRC) patients. The aim of this study was to investigate the association of Bcl-xL expression with invasion and radiosensitivity of human CRC cells.

**Methods:**

RT-PCR and Western blot assays were performed to determine the expression of Bcl-xL mRNA and protein in CRC cells and normal human intestinal epithelial cell line. Then, adenovirus-mediated RNA interference technique was employed to inhibit the expression of Bcl-xL gene in CRC cells. The proliferation of CRC cells was analyzed by MTT and colony formation assay. The migration and invasion of CRC cells was determined by wound-healing and tranwell invasion assays. Additionally, the in vitro and in vivo radiosensitivity of CRC cells was determined by clonogenic cell survival assay and murine xnograft model, respectively.

**Results:**

The levels of Bcl-xL mRNA and protein expression were significantly higher in human CRC cells than in normal human intestinal epithelial cell line. Ad/shBcl-xL could significantly reduce the expression of Bcl-xL protein in CRC cells. Also, we showed that adenovirus-mediated siRNA targeting Bcl-xL could significantly inhibit proliferation and colony formation of CRC cells. Ad/shBcl-xL could significantly suppress migration and invasion of CRC cells. Moreover, Ad/shBcl-xL could enhance in vitro and in vivo radiosensitivity of CRC cells by increasing caspase-dependent apoptosis.

**Conclusions:**

Targeting Bcl-xL will be a promising strategy to inhibit the metastatic potential and reverse the radioresistance of human CRC.

## Introduction

Colorectal cancer, one of the most prevalent cancers in the world, is the second most common malignancy and the second leading cause of cancer related mortality in developed countries [1]. In spite of much progress made in diagnostic and therapeutic methods, the prognosis of CRC patients with distant metastasis still remains poor. Therefore, it is necessary to understand the molecular signaling mechanisms of CRC development so as to provide important insights into more effective therapeutic strategies.

Bcl-xL, an anti-apoptotic member, plays important roles in tumor progression and development [2]. Bcl-xL molecule may inhibit apoptosis by maintaining the permeabilization status or stabilization of the outer mitochondrial membrane [3]. It has been reported that Bcl-xL is overexpressed in many human cancers such as gastric cancer, hepatocelluar cancer, prostate carcinoma, osteosarcoma, breast cancer, etc [4-8]. Previously, we have reported that high level of Bcl-xL protein is correlated with tumor differentiation, lymph node metastasis, venous permeation, and Duke's classification of CRC patients [9]. Furthermore, patients with high Bcl-xL expression showed poorer overall survival than those with low Bcl-xL expression and the status of Bcl-xL protein expression might be an independent prognostic marker for CRC patients. Also, Zhang and his colleagues report that Bcl-xL gene plays an important role in carcinogenesis of human colorectal carcinoma and is associated with malignant biological behaviors of human colorectal carcinoma [10]. Additionally, the correlation between Bcl-xL and chemoresistance of CRC was also reported by other researchers. Guichard' et al showed that short hairpin RNAs targeting Bcl-xL modulated senescence and apoptosis following SN-38 and irinotecan exposure in a colon cancer model [11]. Zhu and his colleagues found that the combination of Bcl-XL-specific small interfering RNA and 5-FU had additive effect on the inhibition of 5-FU-resistant cells [12]. Likewise, Nita'et al showed that the suppression of Bcl-X(L) expression by the specific antisense ODNs could increase the sensitivity of CRC cells to 5-FU [13]. From these experimental data, it was concluded that Bcl-xL might play important roles in the chemoresistance of human CRC. However, whether Bcl-xL affects the metastatic capacity and radiosensitivity of CRC cells is still unclear. To the best of our knowledge, there have been no reports about the correlation between Bcl-xL expression and metastasis or radioresistance of CRC cells.

In the present study, we take advantage of the RNA interference (RNAi) technology, by using an adenoviral construct in order to deliver small interfering RNA molecules that target Bcl-xL gene. RNAi is a highly evolutionarily conserved mechanism of gene regulation, which occurs at a post-transcriptional level. Here, adenovirus-mediated siRNA targeting Bcl-xL could inhibit migration, invasion and metastasis of CRC cells. Meanwhile, Bcl-xL inhibition could increase in vitro and in vivo radiosensitivity of CRC cell lines by increasing apoptotic cell death.

## Materials and methods

### Cell lines

Human colorectal cancer cell lines (SW480, HT-29, LoVo and Colo320) and one human intestinal epithelial cell line (HIEC) were purchased from American Type Culture Collection (ATCC, Manassas, VA). All the cell lines were cultured in Dulbecco's modified Eagle's medium (DMEM) supplemented with 10% fetal calf serum (FCS) and maintained at 37°C in a humidified chamber with 5% CO_2_.

### Recombinant adenovirus generation

The cDNA sequence of Bcl-xL was obtained from GenBank (Accession No. Z23115). The siRNA target design tools from Ambion were used to design shBcl-xL and control shRNA (shcontrol) sequences, which were designed and synthesized as follows: shBcl-xL, sense:5'-*GATCCCCGGAGATGCAGGTATTGGTGttcaagagaCACCAATACCTGCATCTCCTTTT-TGGAAA*-3'; Negative control shRNA, sense: 5'-*GATCCCCGGTGAGAGGTAGGCGTTTAttcaa-gagaTAAACGCCTACCTCTCACCTTTTTGGAAA*-3'; The sequences was subcloned into the *Hin*dIII and *Bgl*II sites of pAdTrack adenoviral shuttle vector to get pAdTrack/shBcl-xLor pAdTrack/shControl. The recombinant vectors were confirmed by the digestion analysis of restriction endonuclease and all inserted sequences were verified by DNA sequencing. The resulting pAdTrack/shBcl-xLor pAdTrack/shControl vectors were linearized with PmeI and co-transformed into BJ5183 cells with adenoviral backbone vector pAdEasy-1. Positive clones were selected and conformed by DNA miniprep and PacI digestion. Plasmids from correct clones were amplified by transforming into DH5K cells. Adenoviral DNA (Ad/shBcl-xL or Ad/shcontrol) was prepared by a standard alkaline lysis procedure, and was linearized with PacI and purified by ethanol precipitation. The packaging cell line 293 was cultured in DMEM with 10% FBS, 100 U/mL penicillin and 100 mg/mL streptomycin. Twenty-four hours before transfection, cells were plated in six-well plates. Cells were transfected with Lipofectamine 2000 plus (Invitrogen, USA). The next day, the medium containing the transfection mix was replaced with fresh medium. Transfected cells were incubated for additional period of 7~10 days and medium was changed every 2~3 days. Virus was harvested, amplified and titered (Stratagene, USA).

### Adenovirus infection

On the day before virus infection, CRC cells were plated in each well of six-well plates. When the cells reached approximately 70-90% confluence, the culture medium was aspirated and the cell monolayer was washed with prewarmed sterile phosphate-buffed saline (PBS). Cells were incubated with indicated virus (Ad/shcontrol or Ad/shBcl-xL) at multiplicity of infection (MOI) of 0, 40 or 80 at 37°C, respectively. After adsorption for 2 h, 2 ml of fresh growth medium was added and cells were placed in the incubator for additional 2-3 days. The cells analysis and other experiments were performed. The following experiments were performed using viruses at such MOIs except for special indications.

### Bcl-xL siRNA oligonucleotide and cell transfection

The siRNA oligonucleotide specific for Bcl-xL (siRNA/Bcl-xL) and scrambled siRNA (siRNA/control) were purchased from Ambion (Austin, TX, USA). Human colorectal cancer cell line (LoVo) was grown in DMEM medium containing 10% fetal calf serum (Gibco BRL, Life Technology, USA) at 37°C in a humidified atmosphere containing 5% CO_2_. Twenty-four hours before transfection, cells were diluted in fresh media without antibiotics and transferred to six-well plates. LoVo cells grown to a confluence of 40%-50% were transfected with 50-200 nmol/L (final concentration) of siRNA per well using Lipofectamine 2000 and Opti-MEM (Invitrogen, Karlsruhe, Germany) media according to the manufacturer's recommendations. 48 h after transfection, the cells were collected for the further researches.

### Reverse Transcription (RT) - PCR assay

Total RNA was extracted from tissues using TRIzol reagent (Invitrogen, Carsbad, CA, USA) according to the manufacturer's instructions. Two micrograms of RNA were subjected to reverse transcription. The PCR primers used were as follows: for Bcl-xL, 5'-*CCCAGAAAGGATACAGC-TGG *-3' (forward), 5'-*GCGATCCGACTCACCAATAC *-3' (reverse); and for GAPDH (internal control), 5'-*GAAGGTGAAGGTCGGATGC*-3' (forward), 5'-*GAAGATGGTGATGGGATTTC*-3' (reverse). The amplification conditions were as follows: denaturation at 94°C for 15 min; 40 cycles of 94°C for 40 s, 56°C (for Bcl-xL) and 60°C (for GAPDH) for 1 min, 72°C for 1 min; and a final 10 min extension at 72°C. The PCR products were separated on a 1.5% agarose gel, visualized, and photographed under UV light.

### Western blot assay

Cells were harvested and washed with cold phosphate-buffered saline solution, and total proteins were extracted in the extraction buffer (150 mM sodium chloride; 50 mMTris hydrochloride, pH7.5;1%glycerol; and1%Non-idetp-40 substitute solution). Equal amounts of protein (15 μg per lane) from the treated cells were loaded and electrophoresed on an 8% sodium dodecyl sulfate (SDS) polyacrylamide gel and then electroblotted onto nitrocellulose membrane, blocked by 5% skim milk, and probed with the antibodies to Bcl-xL, caspase-3 or 9, PARP, uPA and GAPDH (Santa Cruz Biotechnology, Santa Cruz, CA), followed by treatment with secondary antibody conjugated to horseradish peroxidase (1:5000). The proteins were detected by the enhanced chemiluminescence system and exposed to x-ray film.

### Immunohistochemistry assay

Immunohistochemical analysis was done to study altered protein expression in tumor tissues. Formalin-fixed, paraffin-embedded tissue was freshly cut (3 mm). Sections were incubated in a moist chamber with primary rabbit anti-human Bcl-xL monoclonal antibody Santa Cruz Biotechnology, Santa Cruz, CA) for 30 min at room temperature, followed by a secondary antibody (peroxidase labeled polymer conjugated to goat anti-rabbit immunoglobulin) for 30 min (DakoCytomation, Denmark). Rabbit serum was used as negative control.

### 3-(4,5-dimethylthazol-2-yl)-2,5-diphenyltetrazolium bromide (MTT) assay

The cell viability of LoVo cells was measured by a (Sigma, USA). Above three kinds of cells (5.0 × 10^3^/well) were seeded into five 96-well culture plates with each plate having all three kinds of cells (6-parallel wells/group). On each day, 200 μL MTT (5 mg/mL) was added to each well, and the cells were incubated for at 37°C for additional 4 h. Then the reaction was stopped by lysing the cells with 150 μL DMSO for 5 min. Optical densities were determined on a Versamax microplate reader (Molecular Devices, Sunnyvale, CA) at 490 nm.

### Colony formation assay

A total of 4.5 × 10^2 ^mock LoVo or LoVo infected with Ad/shBcl-xL or Ad/shcontrol were placed in a fresh 6-well plate with or without DDP for another 12 h and maintained in RMPI 1640 containing 10% FBS for 2 weeks. Colonies were fixed with methanol and stained with 0.1% crystal violet in 20% methanol for 15 min.

### Wound healing assay

The LoVo cells infected with no adenovirus or adenovirus adenovirus (Ad/shcontrol or Ad/shBcl-xL) at multiplicity of infection (MOI) of 80 were seeded into 24-well tissue culture plates. 48 h later, an artificial homogenous wound was created onto the monolayer with a sterile plastic 100 μL micropipette tip. After wounding, the debris was removed by washing the cells with serum-free medium. Migration of cells into the wound was observed at different time points. Cells that migrated into the wounded area or cells with extended protrusion from the border of the wound were visualized and photographed under an inverted microscope.

### Transwell assay

Transwell invasion experiments were performed with 24-well matrigel-coated chambers from BD Biosciences (Bedford, MA, USA). Briefly, the LoVo cells infected with no adenovirus or adenovirus adenovirus (Ad/shcontrol or Ad/shBcl-xL) at multiplicity of infection (MOI) of 80 were seeded into inserts at 4.0 × 10^3^/insert in serum-free medium and then transferred to wells filled with the culture medium containing 10% FBS as a chemoattractant. After 24 h of incubation, non-invading cells on the top of the membrane were removed by scraping. Invaded cells on the bottom of the membrane were fixed, followed by staining with 0.05% crystal violet. The number of invaded cells on the membrane was then counted under a microscope.

### Flow cytometry analysis of apoptosis

Cells were treated with or without DDP for another 12 h and harvested and fixed with 2.5% glutaraldehyde for 30 minutes. After routine embedment and section, the cells were observed under electronic microscope. The apoptosis rates were determined using Annexin V-FITC and PI staining flow cytometry.

### Clonogenic survival assay

The cells were seeded in 24-well plates. After 24 h-incubation, fresh medium was added to each well and incubation was continued for 24 h before further treatments. One day after the viral infection, cells were trypsinized, plated and incubated for 24 h before irradiation. The time interval between viral infection and radiation treatment was two days. Following irradiation, duplicate cultures were incubated for 10-14 days for colony formation. Cultures were fixed with pure ethanol and stained with 1% crystal violet in ethanol, and colonies were counted. Surviving fraction was determined by normalizing to the plating efficiency of the untreated control cells. For dose fractionation, cells were irradiated with a high-dose rate ^137^Cs unit (4.0 Gy/min) after the viral infections, respectively.

### In vivo radiotherapy assay

BALB/c nude mice were purchased from the Experiment Animal Center of Nanjing Medical University and maintained under pathogen-free conditions according to protocols that were approved by the Jiangsu Province Animal Care and Use Committee. LoVo cells (4.0 × 10^6^) suspended in 100 μl of PBS were inoculated in the flanks of 5-week-old female BALB/c nude mice. One week after inoculation (day 0), mice with established tumors measuring 5-6 mm in diameter, were randomly divided into 5 groups (5 mice/group). Two of the groups were irradiated, and three groups remained unirradiated. For the irradiated groups, intratumoral injections of 0.1 ml of PBS, 6.0 × 10^8 ^pfu of Ad/shcontrol or Ad/shBcl-xL were repeated three times on days 1, 3, and 5, and subsequently X-ray irradiation was performed at a clinically relevant dose of 5.0 Gy on days 2, 4, and 6. For the unirradiated groups, intratumoral injections of 0.1 ml of PBS, 6.0 × 10^8 ^pfu of Ad/shcontrol or Ad/shBcl-xL were administered. Tumors were measured with a caliper gauge twice a week over a 6-week period following the initial virus injections. Tumor volume was calculated according to the formula: TV (mm^3^) = length×width^2 ^×0.4. All mice were sacrificed and s.c tumors were resected and fixed in 10%PBS. We measured the primary tumors and performed Western blot or immunohistochemistry for Bcl-xL protein expression.

### uPA ELISA

Extracts were diluted 1:5 in assay buffer and 100 μL aliquots of each extract were incubated overnight at 4°C in precoated microtest wells. Wells were washed thoroughly with wash buffer and a second, biotinylated antibody that recognizes a specific epitope on uPA molecule was added

for each analysis. Wells were washed again after an incubation of 1 hour and 100 μL of enzyme conjugate was added, leading to the formation of the antibody-enzyme detection complex. After 1 h-incubation, wells were washed again. Then, 100 μL of perborate 3, 3',5, 5'-tetramethylbenzidine substrate was added to each well and reacted with horseradish peroxidase, producing a blue solution. We used 50 μL of 0.5 mol/L sulfuric acid as a stopping solution, which yielded a yellow color in the reaction.

### Statistical analysis

All statistical analyses were performed using the SPSS 17.0 statistical software. Statistical significance of experimental data was determined by Student's t-test (two-tailed). P < 0.05 were deemed statistically significant.

## Results

### The expression level of Bcl-xL is higher in CRC cell lines than in human intestinal epithelial cell line (HIEC)

**Figure 1:**
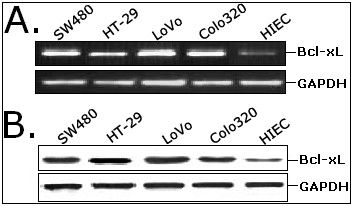
**The expression of Bcl-xL mRNA and protein in four human CRC cell lines (SW480, HT-29, LoVo and Colo320) and one intestinal epithelial cell line (HIEC) by RT-PCR (A) and Western blot assay (B)**. GAPDH was used as an internal control.

Semi-quantitative RT-PCR and Western blot assays were done to determine the expression of Bcl-xL mRNA and protein in four human CRC cell lines (SW480, HT-29, Colo320 and LoVo) and a human intestinal epithelial cell line (HIEC). The expression levels of Bcl-xL mRNA and protein were obviously higher in human CRC cell lines than in human intestinal epithelial cell line (Figure 1A and 1B). Among CRC cell lines, the expression levels of Bcl-xL mRNA and protein shows difference. Meanwhile, in our previous study, we also showed that the average level of Bcl-xL gene in colorectal cancer tissues was significantly higher than that in corresponding nontumor colon tissues at both transcriptional and translational levels. Thus, it was concluded that the overexpression of Bcl-xL might play major roles in CRC tumorigenesis and progression.

### Adenovirus-mediated siRNA targeting Bcl-xL inhibits the expression of Bcl-xL protein in CRC cells

**Figure 2:**
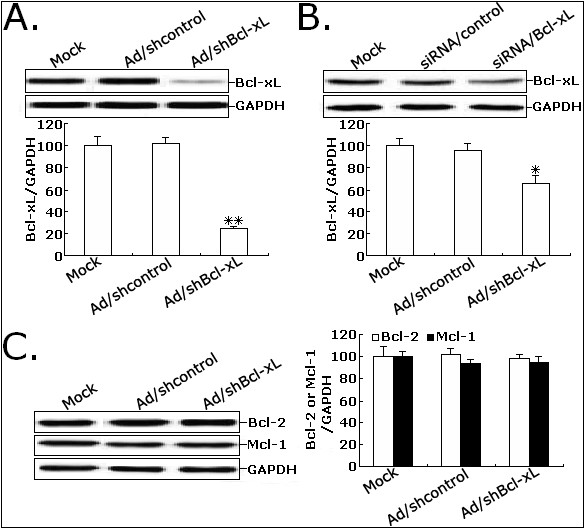
**The expression of Bcl-xL protein in CRC cells was significantly downregualted after adenovirus infection**. A. Western blot analysis of Bcl-xL protein expression in mock LoVo or LoVo infected with Ad/shcontrol or Ad/shBcl-xL. B. Western blot analysis of Bcl-xL protein expression in mock LoVo or LoVo transfected with siRNA/control or siRNA/Bcl-xL. C. Western blot analysis of Bcl-2 and Mcl-1 protein expression in mock LoVo or LoVo infected with Ad/shcontrol or Ad/shBcl-xL. GAPDH was used to an internal control. Each assay was performed at least in triplicate. **P < 0.01 and *P < 0.05 *vs *mock.

To determine the optimal MOI for a maximal transgene expression, LoVo cells were infected with Ad/shcontrol or Ad/shBcl-xl at various MOIs (0, 40, 80) and examined by fluorescence microscopy. Approximately 90% of GFP expression could be observed in LoVo cells infected with Ad/shcontrol or Ad/shBcl-xL at 80 MOI (data not shown). Thus, a MOI of 80 was selected as an optimal dose for infection of CRC cells. To testify the effect of adenovirus-mediated siRNA targeting Bcl-xL on the expression of Bcl-xL gene in CRC cell line, Western blot assay was performed to detect the expression of Bcl-xL protein. The expression level of Bcl-xL protein in Ad/shBcl-xL-infected LoVo cells was decreased by approximately 76.3% by compared to that in mock or Ad/shcontrol-infected cells (*P *< 0.01; Figure 2A). Also, at 48 h after transfection by Lipofectamine 2000, we analyzed the expression of Bcl-xL protein in the siRNA/Bcl-xL-transfected LoVo cells. Compared with mock or siRNA/control-transfected LoVo cells, the expression of Bcl-xL protein was decreased by only 34.6% (*P *< 0.05; Figure 2B). Thus, the knockdown effect of adenovirus-mediated siRNA showed more efficient than that of siRNA oligonucleotide transfected by lipofection. Next, we analyzed the effects of adenovirus-mediated siRNA targeting Bcl-xL on the expression of other apoptosis relevant proteins including Bcl-2 and Mcl-1. As shown in Figure 2C, the levels of Bcl-2 and Mcl-1 protein expression in Ad/shBcl-xL-infected LoVo cells showed no difference compared with those in mock or Ad/shcontrol-infected cells (*P *> 0.05; Figure 2C). These data showed that adenovirus-mediated siRNA targeting Bcl-xL could specifically and significantly inhibit the expression of Bcl-xL gene in CRC cells.

### Ad/shBcl-xL significantly suppresses the proliferation of CRC cells

**Figure 3:**
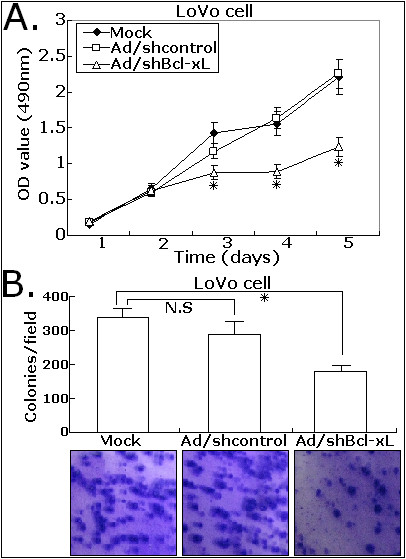
**Effects of Ad/shBcl-xL on the proferation and colony formation of CRC cells**. A. The proliferation of LoVo cells was measured by MTT assay. The cell proliferation of LoVo infected with Ad/shBcl-xL was significantly inhibited in a time-dependent manner. B. The colony number was counted in three different wells at 14 days after seeded, and the averaged number was plotted. The experiments were repeated thrice and similar results were obtained. Representative data are shown. Each assay was performed at least in triplicate. *P < 0.05 *vs *mock.

Given that Ad/shBcl-xL could effectively inhibit Bcl-xL expression, its effects on the proliferation of CRC cells in vitro were determined. As shown in Figure 3A, compared with Ad/shcontrol or mock infection, Ad/shBcl-xL significantly decreased the viability of LoVo cells in a time-dependent manner, and the average proliferation inhibition rates at 3, 4, and 5 days were 39.2%, 42.9%, and 44.3%, respectively (*P *< 0.05). Additionally, Ad/shBcl-xL could significantly reduce the colony formation of LoVo cell by approximately by 35.4% (*P *< 0.01; Figure 3B). These results suggested that siRNA-mediated inhibition of Bcl-xL could suppress the proliferation of CRC cells.

### Ad/shBcl-xL significantly reduces the migration and invasion of CRC cells

**Figure 4:**
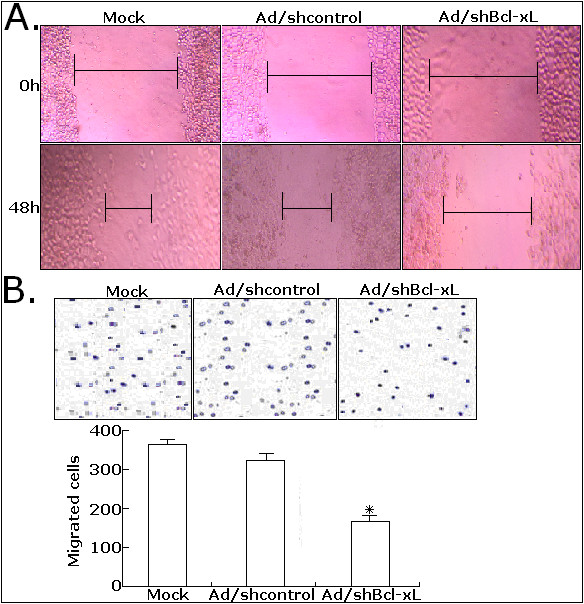
**Effects of Ad/shBcl-xL on migration and invasion of LoVo cells**. A. Scratch wound-healing assay. The cells were infected with mock, Ad/shcontrol or Ad/shBcl-xL as indicated. 72 h after wounding, cells with extended membrane protrusion moved into the wounded areas. Mock: no adenovirus. B. Transwell invasion assay. The invasive LoVo cells were stained and counted under microscope and quantitative results for the transmembrane ability of each group of cells (n = 3). *P < 0.05 *vs *mock.

Prior researches show that Bcl-xL is an anti-apoptotic protein of Bcl-2 family involved in the regulation and promotion of tumor cell survival, but whether Bcl-xL affects the process of tumor invasion and metastasis is still unclear. To investigate the effect of Bcl-xL inhibition on migration and invasion of CRC cells, we performed the scratch-wound and matrigel transwell assays. Scratch-wound assay on confluent monolayers was used as a way of determining cell migration. The mock LoVo or LoVo cells infected with Ad/shcontrol or Ad/shBcl-xL were reseeded in the six-well culture wells with the same cell number for the wound healing assay. At 48 h after wounding, the healing ability of Ad/shBcl-xL-infected LoVo cells significantly lagged behind the mock LoVo or Ad/shcontrol-infected LoVo cells (Figure 4A). Matrigel transwell assay was done to analyze the changes of in vitro invasion capacity of LoVo cells (Figure 4B). Ad/shBcl-xL-infected LoVo cells showed a significantly decreased invasion capacity compared with mock LoVo cells or Ad/shcontrol-infected LoVo cells. A representative experiment demonstrates a marked reduction in the migration of LoVo cells after Ad/shBcl-xL infection compared with mock or Ad/shcontrol infection (*P *< 0.05). The results were quantified from three independent experiments confirming significant decrease in the number of Bcl-xL-ablated LoVo migrating through collagen. These data indicated that adenovirus-mediated siRNA targeting Bcl-xL could significantly inhibit in vitro migration and invasion of CRC cells.

### Ad/shBcl-xL significantly increases the in vitro sensitivity of CRC cells to irradiation

**Figure 5:**
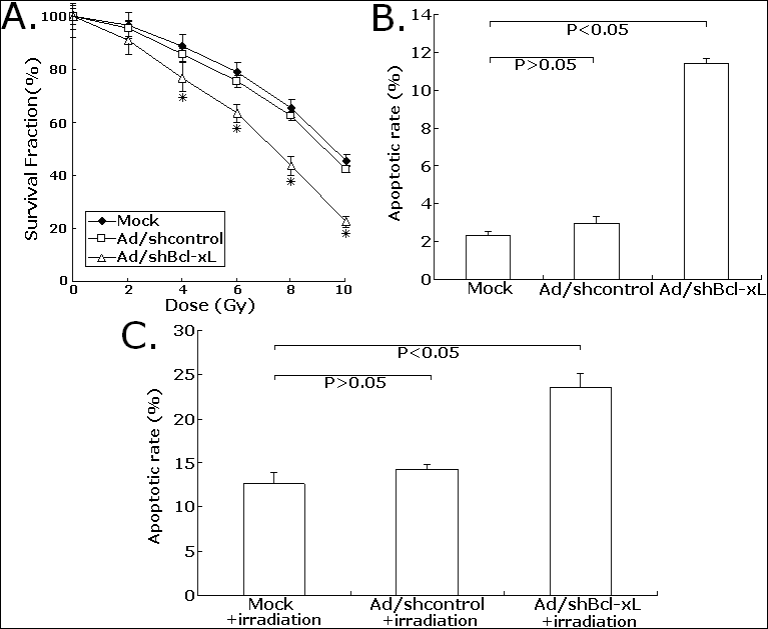
**Effects of Ad/shBcl-xL on the in vitro sensitivity of LoVo cells to irradiation**. A. Clonogenic survival after varying doses of irradiation exposure. The mock, Ad/shcontrol or Ad/shBcl-xL-infected LoVo cells were irradiated followed by a further incubation for 24 h at 37°C before trypsinization and plating for clonogenic survival. After 10-14 days incubation, colonies were stained, and the surviving fraction was determined. The log survival was formed to the number of cells plated, after correcting for plating efficiency. B. Flow cytometry analysis of apoptosis in mock LoVo or LoVo cells infected with Ad/shcontrol or Ad/shBcl-xL. C. Flow cytometry analysis of apoptosis in mock, Ad/shcontrol or Ad/shBcl-xL-infected LoVo combined with irradiation treatment (8.0 Gy). Error bars, the mean ± SD in three experiments (n = 3). *P < 0.05 *vs *mock.

Previously, we have reported that the overexpression of Bcl-xL could affect the sensitivity of osteosarcoma cells to irradiation, but the association of Bcl-xL expression with the radiosensitivity of CRC cells is still unclear. To investigate the radiosensitizing effects of adenovirus-mediated siRNA targeting Bcl-xL on CRC cells, a colony-forming assay was performed. The surviving fraction of the cells infected with Ad/shBcl-xL at a MOI of 80 was significantly lower than that of mock LoVo or Ad/shcontrol-infected LoVo cells at doses of 4.0 and 10.0 Gy (Figure 5A). Then, flow cytometry was performed to analyze the changes of apoptosis in mock LoVo or LoVo cells infected with Ad/shcontrol or Ad/shBcl-xL combined with or without irradiation (8.0 Gy). As shown in Figure 5B, the apoptotic rate of Ad/shBcl-xL-infected LoVo cells was obviously increased by approximately 11.4% compared with mock LoVo cells (*P *< 0.05). The apoptotic rates of mock LoVo or Ad/shcontrol-infected LoVo cells treated with 8.0-Gy irradiation alone were approximately 12.6% and 14.2%, respectively. However, the apoptotic rate of Ad/shBcl-xL-infected LoVo cells increased to 23.6% (*P *< 0.05; Figure 5C). Thus, adenovirus-mediated siRNA targeting Bcl-xL could enhance the radiosensitivity of CRC cells by the increase of radiation-induced apoptosis.

### Ad/shBcl-xL significantly increases the in vivo sensitivity of CRC cells to irradiation

**Figure 6:**
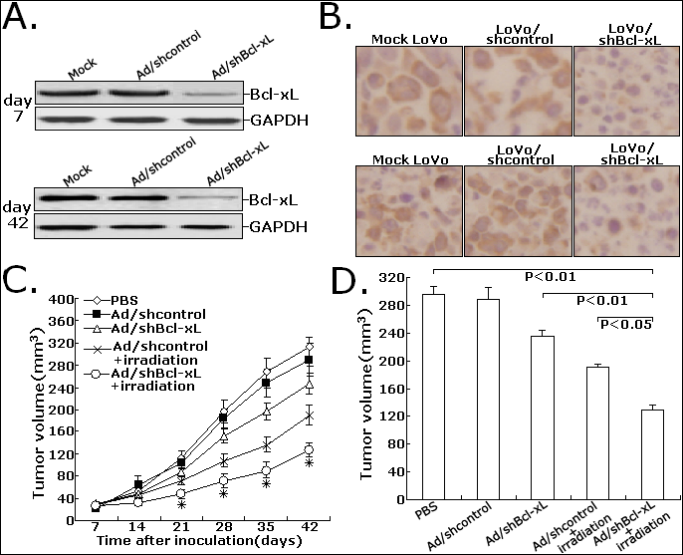
**Effects of Ad/shBcl-xL on the in vivo sensitivity of LoVo cells to irradiation**. A. Protein samples extracted from tumors at day 7 or 42 were determined using Western blot analysis for Bcl-xL expression levels. GAPDH was used as a loading control. B. Immunohistochemistry was performed to detect the expression of Bcl-xL protein in tissues of LoVo tumors treated with PBS, Ad/shcontrol or Ad/shBcl-xL at day 7 or 42. C. Proliferation of tumors in the mice injected with LoVo cells treated with PBS, Ad/shcontrol or Ad/shBcl-xL. D. Dectection of average tumor size at day 42 after the inoculation of LoVo cells treated with PBS, Ad/shcontrol or Ad/shBcl-xL. All experiments were performed in triplicate (n = 3). *P < 0.05 *vs *mock.

Firstly, Western blot assay was performed to detect the expression of Bcl-xL protein in LoVo xenografts on day 7 and 42 post injection of adenovirus. As shown in Figure 6A, the expression of Bcl-xL protein in the turmors in Ad/shBcl-xL-treated group was significantly downregulated compared with PBS or Ad/shcontrol-treated group. Immunohistochemistry assay was performed to analyze the expression of Bcl-xL protein in tumor tissues from LoVo xenografts. As shown in Figure 6B, the tumors in Ad/shBcl-xL-treated group showed significant decreases in the cytoplasmic immunostaining of Bcl-xL protein compared with PBS or Ad/shcontrol-treated group. Next, we attempted to investigate the effect of siRNA-mediated knockdown of Bcl-xL on the in vivo radiosensitivity of CRC cells. Nude mice with established tumors xenografts were treated with PBS or adenovirus (Ad/shBcl-xL or Ad/shcontrol), followed by 4.0Gy-local radiotherapy. A representative growth curve of LoVo xenografted tumors after various treatment was shown in Figure 6C. Ad/shBcl-xL plus irradiation led to a significant suppression of tumor growth compared with Ad/shcontrol plus irradiation or Ad/shBcl-xL alone (*P *< 0.05). On day 42, the tumor-inhibition rates of Ad/shBcl-xL group, Ad/shcontrol plus irradiation group and Ad/shBcl-xL plus irradiation group were 20.2, 35.1 and 56.8%, respectively (*P *< 0.05; Figure 6D). These experimental data showed that adenovirus-mediated siRNA targeting Bcl-xL could increase the in vivo radiosensitivity of CRC cells, which showed that combined Bcl-xL downregulation with radiotherapy could lead to a stronger anti-tumor effect for human CRC.

### Effects of Ad/shBcl-xL on apoptosis or metastasis-related proteins in CRC cells

**Figure 7:**
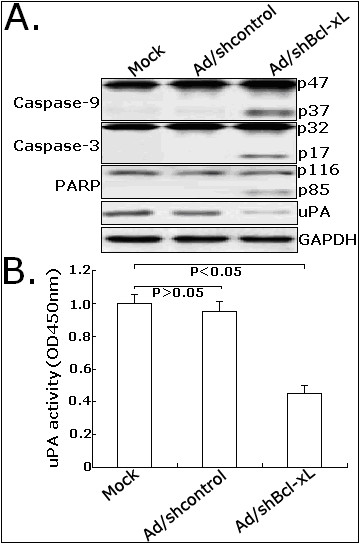
**Effects of Ad/shBcl-xL on the expression of apoptosis or metastasis-related proteins in LoVo cells**. A. Western blot detection of active caspase-9 (p47 and p37), caspase-3 (p32 and p17), PARP (p116 and p85) and uPA protein expression. B. Analysis of activity of uPA in mock LoVo or LoVo cells infected with Ad/shcontrol or Ad/shBcl-xL. The cells in 24-well plates (5.0 × 10^5^/well) were cultured, starved in serum-free medium overnightand. Measurements were made in three separate experiments, and data are shown as mean ± standard deviation. *P < 0.05 *vs *mock. OD: optical density.

The Bcl-2 family members are important regulators of the mitochondrial pathway of apoptosis. Then, we analyzed the effect of siRNA-mediated Bcl-xL inhibition on the expression of caspase-9, caspase-3 and PARP protein. Western blot assay showed that Ad/shBcl-xL could significantly induce activation of caspase-9, caspase-3 and PARP (Figure 7A). Additionally, we showed that Ad/shBcl-xL could significantly inhibit the expression of uPA protein compared with control cells (Figure 7A). Then, we analyzed the changes of uPA activity. As shown in Figure 7B, compared with mock LoVo cells, the activity of uPA in Ad/Bcl-xL-infected LoVo cells was significantly reduced by approximately 54.6% (Figure 7B). Therefore, the changes of those proteins might be involved in Bcl-xL-induced malignant phenotypes of CRC cells.

## Discussion

CRC is the third most commonly diagnosed cancer around the world and the incidence of CRC in China is lower than that in the west countries, but has increased in recent years and become a substantial cancer burden in China, particularly in the more developed areas such as East Guangdong [14]. Despite the current surgical techniques and chemo- or radiotherapy that have made significant improvements, the cure rate for advanced CRC remains low and the morbidity remains high. Therefore, progresses made in CRC therapy might result from a fully understanding of its pathogenesis and biological characteristics.

The Bcl-2 family comprises a group of structurally related proteins that play a fundamental role in the regulation of the intrinsic pathway by controlling mitochondrial membrane permeability and the release of the pro-apoptotic factor, cytochrome c [15]. Usually, those proteins were divided into two classes: those that inhibit apoptosis (Bcl-2, Bcl-xL, Mcl-1, et al); those that promote apoptosis (BAK, Bax, Bcl-xs, et al) [16]. Bcl-xL is an important novel member of anti-apoptotic Bcl-2 family, which has been reported to play critical roles in tumor progression, development and chemo- or radioresistance [17-19]. Tumor metastasis is a highly complex process involving the survival of tumor cells, both in the blood stream and within specific organs [20]. It has been reported that cell-death or survival are determined by a number of gene products from an expanding family of the Bcl-2 gene. In other researches, Bcl-xL was found to be correlated with metastasis of tumor cells. Rubio N and his colleagues reported that overexpression of Bcl-x(L) could counteract the proapoptotic signals in the microenvironment and favor the successful development of metastasis in specific organs [21]. Additionally, Fernández' et al showed that Bcl-xL expression in breast cancer cells could increase metastatic activity and this advantage could be created by inducing resistance to apoptosis against cytokines, increasing cell survival in circulation, and enhancing anchorage-independent growth [22]. Meanwhile, this research group also showed that overexpression of Bcl-xL in human breast cancer cells enhanced organ-selective lymph node metastasis [23]. In our previous study, we showed that the overexpression of Bcl-xL was significantly associated with lymph node metastasis and venous permeation of CRC patients [9]. Zhang and his colleagues also reported that the expression of Bcl-xL was associated with the pathological grade, lymph node metastasis and Duke's stage of colorectal carcinoma [10]. However, the correlation of Bcl-xL expression with invasion and metastasis of CRC cells need to be further elucidated.

RNA interference (RNAi) by double stranded RNA (dsRNAs) molecules of approximately 20-25 nucleotides termed short interfering (siRNAs) is a powerful method for preventing the expression of a particular gene [24]. Plasmid and viral vectors producing siRNA using the polymerase III promoter offer more efficient siRNA delivery and are showing promise both in vitro and in vivo [25]. In our research, adenovirus-mediated siRNA targeting Bcl-xL could significantly inhibit the expression of Bcl-xL in human CRC cells. Adenovirus-mediated siRNA targeting Bcl-xL could significantly inhibit proliferation and colony formation in CRC cells. Moreover, downregulation of Bcl-xL significantly inhibited CRC cell migration in response to wound scratch and impaired the ability of CRC cells to invade across a Matrigel Layer. Subsequently, we investigated the possible molecular mechanisms by which Bcl-xL may enhance tumor invasion. The urinary-type plasminogen activator, or uPA, regarded as the critical trigger for plasmin generation during cell migration and invasion, is strongly upregulated in various malignancies including colorectal cancers [26]. Previously, our and others studies have shown that Bcl-xL is upregulated starting from the earliest stages of dysplasia, we hypothesized that the uPA expression might be related to the dysregulated expression of Bcl-xL in CRC. In our reports, we showed that siRNA-mediated Bcl-xL inhibition could significantly downregulate the expression of uPA protein. The activity of uPA in Ad/shBcl-xL-infected LoVo cells was significantly reduced, which suggested that the Bcl-xL-mediated invasive behavior of CRC cells might be associated with increased activity of uPA. However, how Bcl-xL could affect the expression and activity of uPA in CRC cells still remains unclear and needs to be elucidated in future researches.

Currently, radiotherapy has been an integral part of the preoperative treatment of rectal cancers. However, only a minority of patients achieve a complete pathologic response to therapy because of radioresistance of these tumors [27]. Previously, we have shown that the overexpression of Bcl-xL is associated with radioresistance of human osteosarcoma cells. In other human cancers, the association of Bcl-xL expression and radiosensitivity of tumor cells is also studied. Wang' et al showed that siRNA-mediated Bcl-xL downregulation could lead to the increased sensitivity of prostate cancer cells to radiation [28]. Masui, et al showed that the antisense oligonucleotide against Bcl-XL could be a good therapeutic tool for radiosensitization of pancreatic cancer [29]. Streffer and his colleagues showed that BCL-2 family protein expression (Bcl-xL and BAX) could modulate radiosensitivity in human glioma cells and targeted alterations in BCL-2 family protein expression might be a promising strategy to improve the therapeutic efficacy of radiotherapy for gliomas [30]. However, up to date, there have been no reports about the associations of Bcl-xL expression and radiosensitivity of human CRC cells. In this study, Ad/shBcl-xL could increase the radiosensitivity of CRC cells not only in vitro but also in vivo, which might be mainly associated with caspase-dependent apoptosis enhancement.

In conclusion, we have shown for the first time that adenovirus-mediated siRNA targeting Bcl-xL could inhibit proliferation and enhance radiosensitivity of human CRC cells. Moreover, our results also showed that Bcl-xL played an important role in CRC cell invasion, which might be mediated by uPA. Therefore, Bcl-xL has potential of being a novel molecular target for the treatment of human CRC.

## Competing interests

The authors declare that they have no competing interests.

## Authors' contributions

JY supervised research project and drafted the manuscript. MS participated in cell culture. AZ and CL carried out the operation and participated in the data collection. WD and ZW acted as corresponding author and made the revisions. All authors read and approved the final manuscript.
